# Carbon Dioxide Emissions as Affected by Alternative Long-Term Irrigation and Tillage Management Practices in the Lower Mississippi River Valley

**DOI:** 10.1155/2014/626732

**Published:** 2014-10-13

**Authors:** S. F. Smith, K. R. Brye

**Affiliations:** Department of Crop, Soil, and Environmental Sciences, University of Arkansas, Fayetteville, AR 72701, USA

## Abstract

Ensuring the sustainability of cultivated soils is an ever-increasing priority for producers in the Lower Mississippi River Valley (LMRV). As groundwater sources become depleted and environmental regulations become more strict, producers will look to alternative management practices that will ensure the sustainability and cost-effectiveness of their production systems. This study was conducted to assess the long-term (>7 years) effects of irrigation (i.e., irrigated and dryland production) and tillage (conventional and no-tillage) on estimated carbon dioxide (CO_2_) emissions from soil respiration during two soybean (*Glycine max* L.) growing seasons from a wheat- (*Triticum aestivum* L.-) soybean, double-cropped production system in the LMRV region of eastern Arkansas. Soil surface CO_2_ fluxes were measured approximately every two weeks during two soybean growing seasons. Estimated season-long CO_2_ emissions were unaffected by irrigation in 2011 (*P* > 0.05); however, during the unusually dry 2012 growing season, season-long CO_2_ emissions were 87.6% greater (*P* = 0.044) under irrigated (21.9 Mg CO_2_ ha^−1^) than under dryland management (11.7 Mg CO_2_ ha^−1^). Contrary to what was expected, there was no interactive effect of irrigation and tillage on estimated season-long CO_2_ emissions. Understanding how long-term agricultural management practices affect soil respiration can help improve policies for soil and environmental sustainability.

## 1. Introduction

Soybean (*Glycine max* L.) is a major crop grown in the Lower Mississippi River Valley (LMRV). Arkansas produces the greatest amount of soybean of the three states that generally constitute the LMRV (i.e., Arkansas, Louisiana, and Mississippi) and is ranked eighth nationally for total economic gain from soybean production [1]. The Alluvial Aquifer, which is the southeastern portion of the Mississippi embayment, is the major source of groundwater used for irrigation in this dense, agriculturally productive region. The Alluvial Aquifer is ranked third in the nation for total annual withdrawals [2], and most of the water is used for irrigated crop production, particularly rice (*Oryza sativa* L.) and soybean [3]. However, increased withdrawal rates from the Alluvial Aquifer, due in part to increasing areas of irrigated rice and soybean production and shifting rainfall patterns during the growing season, have led to substantial decreases in groundwater levels throughout eastern Arkansas and the neighboring states [4].

In order to assure adequate soybean yields, most producers irrigate during the growing season on an as-needed basis [5, 6]. Alternatively, when water is unavailable or the implementation of irrigation is too costly, producers will practice dryland production, in which the sole source of water to the crop is rainfall. Recently, 20% of soybean produced in eastern Arkansas was grown in a dryland cropping system [7]. However, irrigation can be absolutely essential to producing optimal yields to meet economic demands, especially in a wheat-soybean, double-crop system where soybean is planted in late spring. During warm weather, the lack of available water in a dryland cropping system can result in yield loss [6]. Consequently, it is important for producers to choose management options that can help conserve soil water and protect the crop from water stress.

Conventional tillage (CT) is the most common soil management practice in the LMRV region of eastern Arkansas. Conventional tillage prior to a soybean growing season in the region generally consists of several passes with a disk to manipulate a soil depth of 5 to 10 cm, followed by smoothing with a soil conditioner to break up soil clods. However, CT breaks up the soil's natural structure and leaves the soil surface nearly bare and vulnerable to wind and water erosion at times [8]. Following CT, weakened soil structural stability near the surface can increase the risk of the formation of a surface crust or seal, which can further reduce water infiltration and the soil's water-holding capacity [9]. It has been documented that the long history of cultivated agriculture in the LMRV region of eastern Arkansas has severely impacted infiltration-runoff partitioning [10]. The impacts have been so drastic that the recharge area for the Alluvial Aquifer has been condensed and shifted to the point where annual recharge of the Alluvial Aquifer is only a small fraction of the annual withdrawals for irrigation [10].

As an alternative to CT, producers can practice no-tillage (NT) in order to maximize water conservation and improve many soil-quality-related characteristics [9, 11]. When NT is used, maintaining surface residue can improve soil water retention by reducing runoff and by acting as a protective and evaporative barrier for the soil surface. As water becomes increasingly limited, improving soil-quality-related characteristics and using management practices that promote water conservation will likely become increasingly necessary to ensure sustained yields.

A good indicator of the soil's ability to support plant life is soil respiration, which is the combined production of carbon dioxide (CO_2_) from soil organisms and plant roots. Soil respiration is closely correlated with soil organic matter content, root respiration, microbial activity, and decomposition rates [12]. Soil respiration can be influenced by many environmental conditions, such as moisture and temperature [13]. Generally, optimal conditions for soil respiration occur when the soil is warm and the soil water content is near field capacity.

Environmental factors affecting soil respiration can be manipulated by residue- and water-management practices in agroecosystems. In general, management practices that promote plant biomass formation (e.g., adequate soil fertility), increase the bioavailability of carbon (C) sources (e.g., tillage), and maintain optimal soil moisture for soil microbial activity (e.g., irrigation) will increase soil respiration rates [13]. Inferences about nutrient cycling can also be made using soil respiration rates. Excessive respiration can indicate that a soil is losing nutrients or is in a state of flux following a disturbance, such as tillage. In contrast, decreased soil organic matter, poor soil structure, and limited nutrient availability may inhibit soil respiration and indicate poor soil quality. Root respiration can account for up to 80% of total soil CO_2_ emissions [12]; therefore, plant biomass production and productivity that has been limited from water-stressed conditions can result in greatly reduced soil respiration rates and seasonal CO_2_ emissions.

Since CO_2_ is a greenhouse gas of concern and atmospheric concentrations have increased dramatically in the past half century or so [14], there is a growing worldwide interest to identify ways to reduce CO_2_ emissions, sequester C, and remove CO_2_ from the atmosphere. Optimizing agroecosystem management practices for soil sustainability can also help store C from the atmosphere in a semipermanent state [15]. Furthermore, there are few studies that have examined the impacts of long-term (>5 years) agricultural management practices on soil respiration, especially in the midsouthern United States. Therefore, the objective of this study was to evaluate the effects of irrigation (irrigated and dryland), tillage (conventional tillage (CT) and no-tillage (NT)), and residue level (high and low) on season-long CO_2_ emissions from soil respiration after more than seven years of consistent management in a wheat-soybean, double-crop production system in the LMRV region of eastern Arkansas. It was hypothesized that irrigation would increase CO_2_ emissions by reducing the occurrence of water-stressed conditions, especially during warm and dry periods. It was also hypothesized that irrigation would mask any differential effects of tillage on CO_2_ emissions and that differential effects of tillage on CO_2_ emissions would be greater in dryland production.

## 2. Materials and Methods

### 2.1. Site Description

This long-term study was initiated in fall 2001 at the University of Arkansas' Lon Mann Cotton Research Station (N 34°, 44′, 2.26′′ and W 90°, 45′, 51.56′′), which is located in east-central Arkansas, near Marianna in Lee County [16]. The study was conducted on a Calloway silt loam (fine-silty, mixed, active, thermic Aquic Fraglossudalf) [17].

The 30-year mean monthly air temperatures for the soybean growing season for the region are 25.4, 27.3, 26.3, 22.7, and 16.8°C for June, July, August, September, and October, respectively [18]. The 30-year monthly rainfall amounts for the soybean growing season for the region are 11.1, 9.7, 6.9, 8.0, and 9.6 cm for June, July, August, September, and October, respectively [18]. The 30-year mean annual air temperature in the region is 15.9°C and the mean annual precipitation is 133.7 cm [18].

### 2.2. Treatments and Experimental Design

A randomized complete block design, consisting of burning, tillage, and fertility treatments, with three replications was established in 2001 [16]. Two replications of the burning treatment were arranged as a randomized complete block (RCB). Three replications of tillage were also arranged as a RCB but were stripped across the burning block. The fertility treatments were split into each burn-tillage combination. The whole field was split in half in 2005 in order to incorporate the irrigation treatment. Due to practical limitations, the irrigation treatment was necessarily placed in the experimental design with a similar blocking structure as the burning treatment. This resulted in three replications for each of the 16 possible treatment combinations. The irrigation treatment consisted of either irrigating the field on an as-needed basis (irrigated) or no irrigation applied during the soybean growing season (dryland). The tillage treatment was a strip within the irrigation treatment and consisted of CT or direct sowing of soybean after harvest with no residue incorporation (i.e., NT). A nitrogen (N) fertility treatment, used to produce differing amounts of wheat residue into which the subsequent soybean crop would be planted, was established as a split plot within the tillage treatment. The study site consisted of 48 plots, each 3 m wide and 6 m long [16].

### 2.3. Field Management

Following the application of the tillage treatment each year, a glyphosate-resistant soybean variety of maturity group 5.3 to 5.4 was planted with a 19-cm row spacing throughout the study site in early-to-mid-June. Soybean grown within the irrigated treatment was irrigated on an as-needed basis [6], while the dryland soybean was only rain fed. Soybean harvest generally occurred between late October and the middle of November each year. Following soybean harvest, wheat was drill-seeded with 19-cm row spacing [19]. Since 2005, wheat grown within the high-fertility treatment was fertilized with a split application of urea (i.e., 56 kg N ha^−1^ applied in early March and 56 kg N ha^−1^ in late March), while a low-fertility treatment received no N applications. In early-to-mid-June each year, wheat was harvested and the remaining stubble was mowed with a rotary mower to create a uniform layer of residue. After mowing and prior to tillage, a residue burning treatment was imposed each year [20]. Additional details regarding the history of the study site and past management are summarized in Brye et al. [19], Cordell et al. [16], and Smith et al. [20].

### 2.4. Soil Respiration Measurements

During the 2011 and 2012 soybean growing seasons (i.e., June to October), soil respiration was measured approximately every two weeks in the morning between 0600 and 1100 hours. There were a total of 124 and 151 days in the 2011 and 2012 soybean growing seasons, respectively. Respiration measurements were conducted nine times in 2011 and 11 times in 2012. At least one day prior to measurements, 10-cm diameter PVC collars, with a beveled edge on the bottom, were placed in each plot to facilitate soil respiration measurements, similar to the procedures used in a previous study [19]. A portable infrared gas analyzer (LI-6400, LI-COR, Inc., Lincoln, NE) with a soil chamber attachment (LI-6400-09, LI-COR) was used as per manufacturer's recommendations and as used previously [19].

### 2.5. Soybean Yields

In late October each year, soybean from the middle 1.5 m of each plot was harvested with a plot combine. The harvest area was 1.5-m wide and 6-m long. Grain was air-dried for approximately three weeks before weighing. A subsample from each plot was oven dried for 48 hr at 70°C and weighed to adjust reported yields to a 13% moisture content.

### 2.6. Data Analyses

Growing-season-long CO_2_ emissions were estimated using linear interpolation between measurement dates and the trapezoid method was used for area-under-the-curve calculations [21, 22]. Since irrigation was superimposed into the experimental design with a similar blocking structure as the burning treatment, the two treatments were confounded. Therefore, irrigation and burning treatments could not be analyzed together. For this reason, two separate three-factor analyses of variance (ANOVAs) were conducted based on a strip-split-plot design, each excluding one of the confounding factors. The PROC GLM procedure in SAS (version 9.2 SAS Institute, Inc., Cary, NC) was used to evaluate the effects of burning or irrigation, tillage, fertility, year, and their interactions on season-long CO_2_ emissions. This study was focused on the effects of irrigation and any interactions containing irrigation on soil respiration. For the results concerning the effects of burning and interactions containing burning, see Smith et al. [20]. Where appropriate, means were separated by least significant difference (LSD) at the 0.05 level.

## 3. Results and Discussion

### 3.1. Growing-Season Weather Conditions

The growing-season weather conditions the soybean crop experienced differed somewhat between 2011 and 2012 and differed somewhat from the 30-year mean conditions. The 30-year mean monthly air temperature in eastern Arkansas near Marianna peaks at 27.3°C in July decreases thereafter to 16.8°C in October and averages 23.7°C throughout the typical 5-month soybean growing season [18]. The 30-year monthly rainfall in eastern Arkansas ranges from 6.9 cm in August to 11.1 cm in June and totals 45.3 cm throughout the 5-month soybean growing season [18]. Actual mean air temperatures measured on-site in 2011 (24.3°C) and 2012 (24.2°C) throughout the 5-month growing season were only slightly greater than the 30-year mean air temperature for the same 5-month period. However, for both 2011 (38.5 cm) and 2012 (35.4 cm), rainfall amounts measured on-site were at least 15% lower than the 30-year mean rainfall total (45.3 cm) [18] over the 5-month soybean growing season. Furthermore, eastern Arkansas experienced a drought in 2012 relative to previous years. Though total rainfall during the 5-month soybean growing season was only 8% lower in 2012 than in 2011, total rainfall in June, July, and August, the critical early period of the soybean crop, in 2012 (11.6 cm) was less than 43% of that in 2011 (27.2 cm) and less than 42% of the 30-year mean total rainfall (27.7 cm) [18]. Based on differences in growing-season rainfall, both seasonal CO_2_ emissions and soybean yields were expected to differ between 2011 and 2012.

### 3.2. Seasonal CO_2_ Emissions

Estimated season-long CO_2_ emissions from soil respiration, measured 20 times throughout the 2011 and 2012 soybean growing seasons in eastern Arkansas, differed between irrigation treatments across years (*P* = 0.044) and differed between tillage treatments (*P* = 0.020; Table 1). Season-long CO_2_ emissions averaged 22.3 Mg CO_2_ ha^−1^ in 2011 and were unaffected by the irrigation treatments (irrigated (22.4 Mg CO_2_ ha^−1^) and dryland (22.2 Mg CO_2_ ha^−1^). However, estimated season-long CO_2_ emissions were 87.6% greater under irrigated (21.9 Mg CO_2_ ha^−1^) compared to under dryland management (11.7 Mg CO_2_ ha^−1^) in the drought year of 2012.

Total season-long emissions were closely tied with soybean yields. During the more favorable 2011 growing season, soybean yields were 3.2 and 1.7 Mg ha^−1^ for the irrigated and dryland treatments, respectively. However, only 0.15 Mg ha^−1^ of soybean was harvested from the dryland treatment during the drier 2012 year, which was 6% of the soybean yield from the irrigated treatment (2.5 Mg ha^−1^).

Although season-long CO_2_ emissions may be limited by water-stressed conditions, a substantial pulse of CO_2_ from the soil can follow a precipitation or irrigation event when soil microbial activity is stimulated after a long dry period [23]. Peak respiratory pulses on the order of 60 to 80 times the baseline respiration rate were reported following rainfall events in an annual grassland and a nearby oak- (*Quercus douglasii-*) grass savanna on a rocky silt-loam soil in California [23]. Sainju et al. [24] concluded that antecedent soil water content and water retention were the two greatest determinants for soil CO_2_ pulse intensities and duration following rainfall or irrigation in a North Dakota barley- (*Hordeum vulgare *L.-) pea (*Pisum sativum *L.) rotation. Although many others have described similar soil CO_2_ pulses after irrigation and rainfall events [25–27], the intensities and durations have been extremely variable, even within the same study area [28].

Irrigation can have a direct effect on soil respiration by regulating available soil water for microbial and plant activity. Optimal soil moisture for plant and microbial function is generally around field moisture capacity, where micropores (i.e., <0.08 mm diameter) in the soil are still mostly filled with water and macropores (i.e., >0.08 mm diameter) are mostly air-filled, which facilitates oxygen diffusion [12]. Below field moisture capacity, water and nutrients can become limiting for plants and microorganisms. When biological consumption of soil organic matter is limited, soil C can build up in the soil. Although plant productivity may be limited in dry years, dryland agriculture can have both economic and environmental benefits [29].

Mean annual declines in the Alluvial Aquifer's water levels between 1982 and 2006 have been reported to be around 16.8 cm yr^−1^ for Lee County, Arkansas [4]. By using the recharge rate and rate of withdrawal in 1997, Scott et al. [30] estimated that the Alluvial Aquifer's water available for irrigation would be depleted by 2050. Since water is becoming more limited and since some agricultural production is on land that is difficult to irrigate, some producers choose to rely only on rainfall to water their soybean crop. During favorable years, dryland management can save producers the cost of installation of the irrigation system and water-pumping costs throughout the season. Although this is a bit of a gamble with natural precipitation being hard to predict, dryland soybean production can work in the producer's favor. Parsch et al. [31] reported that nonirrigated soybean grown in eastern Arkansas generally provided a larger net economic return for the producer than irrigated soybean. Verkler et al. [29] also demonstrated that, during a favorable rainfall year, dryland soybean production was equally profitable as irrigated soybean production in eastern Arkansas. However, not surprisingly, during growing seasons of less-than-optimal rainfall, dryland production was much less profitable than irrigated soybean production. Results of this study indicate that dryland soybean production may be more environmentally sustainable due to the lower water consumption and generally lower seasonal CO_2_ emissions.

Beyond the potential economic value that can be associated with dryland cropping, there are numerous environmental benefits. In addition to preserving groundwater, dryland cropping can favor the accumulation of soil organic matter by reducing erosion of topsoil and/or by decreasing soil respiration. Depending on the irrigation system implemented, soil erosion can occur due to the sudden inundation of fragile soil aggregates, which can disintegrate upon submersion, raindrop impact from sprinkler systems, and/or surface erosion caused by runoff in surface irrigation systems [32]. Churchman and Tate [33] reported a decrease in total water-stable aggregates and soil C stocks after >25 years of annual irrigation of a seasonally dry, silt-loam soil in New Zealand, which was likely due to increased microbial decomposition compared to dryland production. The sudden inundation of the soil by water from furrow irrigation, the most common soybean irrigation technique in eastern Arkansas, can also cause slaking to occur and unstable aggregates to disintegrate. The most unstable, larger aggregates are more affected by slaking due to irrigation than the aggregates that are smaller in diameter [34]. In addition, near-surface soil aggregates, many of which protect soil organic matter and C, are greatly impacted by mechanical disturbances, such as tillage [34].

Although many others have noted a short-term increase in soil respiration following tillage [21, 34–37], we expected these fluctuations to remain short in duration and likely not persist throughout an entire growing season. Wander and Bidart [11] reported greater CO_2_ fluxes following tillage; however, the increased rates of soil surface CO_2_ evolution only lasted approximately 24 hours. No-tillage, which has been shown to improve soil water conservation [38], increase soil organic matter and C contents [11], and improve soil structure [39], was expected to produce greater season-long CO_2_ than CT, especially within the dryland treatment. However, in contrast to that hypothesized, CT produced greater season-long CO_2_ than NT, regardless of irrigation scheme. When averaged over all other field treatments and years, estimated season-long CO_2_ emissions were 21.0 Mg CO_2_ ha^−1^ yr^−1^ under CT, compared to only 18.1 Mg CO_2_ ha^−1^ yr^−1^ under NT (*P* = 0.020; Table 1). Although production of CO_2_ may indicate a healthy soil with high microbial activity, some soils that have historically low concentrations of organic matter to sustain microbial activity may benefit from lower decomposition rates. Since the study area is in a region with generally low soil organic matter concentrations (~2.1% by loss-on-ignition; [40]) due to a long history of highly productive, cultivated agriculture, producers may benefit from using NT management practices that reduce the amount of C emitted to the atmosphere via soil respiration in both irrigated and dryland cropping systems.

## 4. Conclusions

The impact of irrigation on seasonal CO_2_ emissions differed between years. As was expected, irrigation only affected estimated season-long CO_2_ emissions during water-stressed conditions by supplementing adequate soil moisture for soybean growth and soil microbial respiration. Contrary to that hypothesized, the tillage effect on total CO_2_ emissions was not dependent on the irrigation scheme used. No-tillage management reduced seasonal CO_2_ emissions during both soybean growing seasons.

## Figures and Tables

**Table 1:** Analysis of variance summary of the effects of irrigation, tillage, residue level (residue), year, and their interactions on estimated season-long CO_2_ emissions at the University of Arkansas' Lon Mann Cotton Research Station near Marianna, AR, on a silt-loam soil. Interactions and main effects that are considered significant are indicated by bolded text (*P* < 0.05). This analysis ignores the burning treatment in the design.

Treatment effect	Season-long
	CO_2_ emissions
Irrigation	0.243
Tillage	**0.020**
Irrigation ∗ tillage	0.383
Residue	0.512
Irrigation ∗ residue	0.097
Tillage ∗ residue	0.699
Irrigation ∗ tillage ∗ residue	0.759
Year	**<0.001**
Irrigation ∗ year	**0.044**
Tillage ∗ year	0.366
Residue ∗ year	0.689
Irrigation ∗ tillage ∗ year	0.277
Irrigation ∗ residue ∗ year	0.926
Tillage ∗ residue ∗ year	0.455
Irrigation ∗ tillage ∗ residue ∗ year	0.838

## References

[1] USDA-ERS State fact sheets: arkansas. http://www.ers.usda.gov/data-products/state-fact-sheets.aspx.

[2] Maupin M. A., Barber N. L. (2005). *Estimated Withdrawals from Principal Aquifers in the United States, 2000*.

[3] Clark B. R., Hart R. M., Gurdak J. J. (2011). *Groundwater Availability of the Mississippi Embayment*.

[4] Schrader T. P. (2008). *Water Levels and Selected Water-quality Conditions in the Mississippi River Valley Alluvial Aquifer in Eastern Arkansas, 2006*.

[5] Bajaj S., Chen P., Longer D. E., Shi A., Hou A., Ishibashi T., Brye K. R. (2008). Irrigation and planting date effects on seed yield and agronomic traits of early-maturing soybean. *Journal of Crop Improvement*.

[6] UACES (2000). *Arkansas Soybean Handbook*.

[7] USDA-NASS Statistics by state: arkansas. http://www.nass.usda.gov/Statistics_by_State/Arkansas/index.asp.

[8] Six J., Elliott E. T., Paustian K. (2000). Soil macroaggregate turnover and microaggregate formation: a mechanism for C sequestration under no-tillage agriculture. *Soil Biology and Biochemistry*.

[9] Pikul Jnr J. L., Zuzel J. F. (1994). Soil crusting and water infiltration affected by long-term tillage and residue management. *Soil Science Society of America Journal*.

[10] Harper T. W., Brye K. R., Daniel T. C., Slaton N. A., Haggard B. E. (2008). Land use effects on runoff and water quality on an Eastern Arkansas soil under simulated rainfall. *Journal of Sustainable Agriculture*.

[11] Wander M. M., Bidart M. G. (2000). Tillage practice influences on the physical protection, bioavailability and composition of particulate organic matter. *Biology and Fertility of Soils*.

[12] Luo Y., Zhou X. (2010). *Soil Respiration and the Environment*.

[13] Franzluebbers A. J., Hons F. M., Zuberer D. A. (1995). Tillage and crop effects on seasonal soil carbon and nitrogen dynamics. *Soil Science Society of America Journal*.

[14] Jones N. (2013). Troubling milestone for CO_2_. *Nature Geoscience*.

[15] Kirschbaum M. U. F. (2000). Will changes in soil organic carbon act as a positive or negative feedback on global warming?. *Biogeochemistry*.

[16] Cordell M. L., Brye K. R., Longer D. E., Gbur E. E. (2007). Residue management practice effects on soybean establishment and growth in a young wheat-soybean double-cropping system. *Journal of Sustainable Agriculture*.

[17] NRCS Web Soil Survey. Natural Resources Conservation Service. http://websoilsurvey.sc.egov.usda.gov/App/HomePage.htm.

[18] NOAA (2002). *Climatography of the United States No. 81, Monthly Station Normals of Temperature, Precipitation, and Heating and Cooling Degrees Days 1971–2000: Arkansas*.

[19] Brye K. R., Longer D. E., Gbur E. E. (2006). Impact of tillage and residue burning on carbon dioxide flux in a wheat-soybean production system. *Soil Science Society of America Journal*.

[20] Smith F., Brye K. R., Gbur E. E., Chen P., Korth K. (2014). Long-term residue management effects on soil respiration in a wheat-soybean double-crop system. *Soil Science*.

[21] Reicosky D. C. (1997). Tillage-induced CO_2_ emission from soil. *Nutrient Cycling in Agroecosystems*.

[22] Morell F. J., Cantero-Martínez C., Lampurlanés J., Plaza-Bonilla D., Álvaro-Fuentes J. (2011). Soil carbon dioxide flux and organic carbon content: Effects of tillage and nitrogen fertilization. *Soil Science Society of America Journal*.

[23] Xu L., Baldocchi D. D., Tang J. (2004). How soil moisture, rain pulses, and growth alter the response of ecosystem respiration to temperature. *Global Biogeochemical Cycles*.

[24] Sainju U. M., Jabro J. D., Stevens W. B. (2008). Soil carbon dioxide emission and carbon content as affected by irrigation, tillage, cropping system, and nitrogen fertilization. *Journal of Environmental Quality*.

[25] Verma S. B., Dobermann A., Cassman K. G., Walters D. T., Knops J. M., Arkebauer T. J., Suyker A. E., Burba G. G., Amos B., Yang H., Ginting D., Hubbard K. G., Gitelson A. A., Walter-Shea E. A. (2005). Annual carbon dioxide exchange in irrigated and rainfed maize-based agroecosystems. *Agricultural and Forest Meteorology*.

[26] Jabro J. D., Sainju U., Stevens W. B., Evans R. G. (2008). Carbon dioxide flux as affected by tillage and irrigation in soil converted from perennial forages to annual crops. *Journal of Environmental Management*.

[27] Bauder J., Schneider R. (1979). Nitrate-nitrogen leaching following urea fertilization and irrigation. *Soil Science Society of America Journal*.

[28] Rochette P., Desjardins R. L., Pattey E. (1991). Spatial and temporal variability of soil respiration in agricultural fields. *Canadian Journal of Soil Science*.

[29] Verkler T. L., Brye K. R., Popp J. H., Gbur E. E., Chen P., Amuri N. (2009). Soil properties, soybean response, and economic return as affected by residue and water management practices. *Journal of Sustainable Agriculture*.

[30] Scott H. D., Ferguson J. A., Hanson L., Fugitt T., Smith E. (1998). *Agricultural Water Management in the Mississippi Delta Region of Arkansas*.

[31] Parsch L. D., Keisling T. C., Sauer P. A., Oliver L. R., Crabtree N. S. (2001). Economic analysis of conservation and conventional tillage cropping systems on clayey soil in eastern Arkansas. *Agronomy Journal*.

[32] Lehrsch G., Bjorneberg D., Sojka R. (2005). Erosion: irrigation-induced. *Encyclopedia of Soils in the Environment*.

[33] Churchman G., Tate K. (1986). Effect of slaughterhouse effluent and water irrigation upon aggregation in seasonally dry New Zealand soil under pasture. *Soil and Tillage Research*.

[34] Six J., Paustian K., Elliott E. T., Combrink C. (2000). Soil structure and organic matter I. Distribution of aggregate-size classes and aggregate-associated carbon. *Soil Science Society of America Journal*.

[35] Gesch R. W., Reicosky D. C., Gilbert R. A., Morris D. R. (2007). Influence of tillage and plant residue management on respiration of a Florida Everglades Histosol. *Soil & Tillage Research*.

[36] Dong W., Hu C., Chen S., Zhang Y. (2009). Tillage and residue management effects on soil carbon and CO_2_ emission in a wheat-corn double-cropping system. *Nutrient Cycling in Agroecosystems*.

[37] Ellert B. H., Janzen H. H. (1999). Short-term influence of tillage on CO_2_ fluxes from a semi-arid soil on the Canadian Prairies. *Soil and Tillage Research*.

[38] Dabney S. M. (1998). Cover crop impacts on watershed hydrology. *Journal of Soil and Water Conservation*.

[39] Kasper M., Buchan G. D., Mentler A., Blum W. E. H. (2009). Influence of soil tillage systems on aggregate stability and the distribution of C and N in different aggregate fractions. *Soil and Tillage Research*.

[40] DeLong R., Slaton N., Brye K., Wolf N., Mosaffari M. Relationships between organic carbon and other chemical properties in Arkansas soils.

